# Photo Quiz: Asteroid bodies in a skin biopsy of a farmer

**DOI:** 10.1128/jcm.00478-25

**Published:** 2025-07-09

**Authors:** Xiujiao Xia

**Affiliations:** 1 Department of Dermatology, Hangzhou Third People’s Hospital, Hangzhou Third Hospital Affiliated to Zhejiang Chinese Medical University 70571, Hangzhou, Zhejiang, China; Mayo Clinic Minnesota, Rochester, Minnesota, USA

**Keywords:** *Sporothrix globosa*, asteroid body, sporotrichosis

## PHOTO QUIZ 

**Fig 1:**
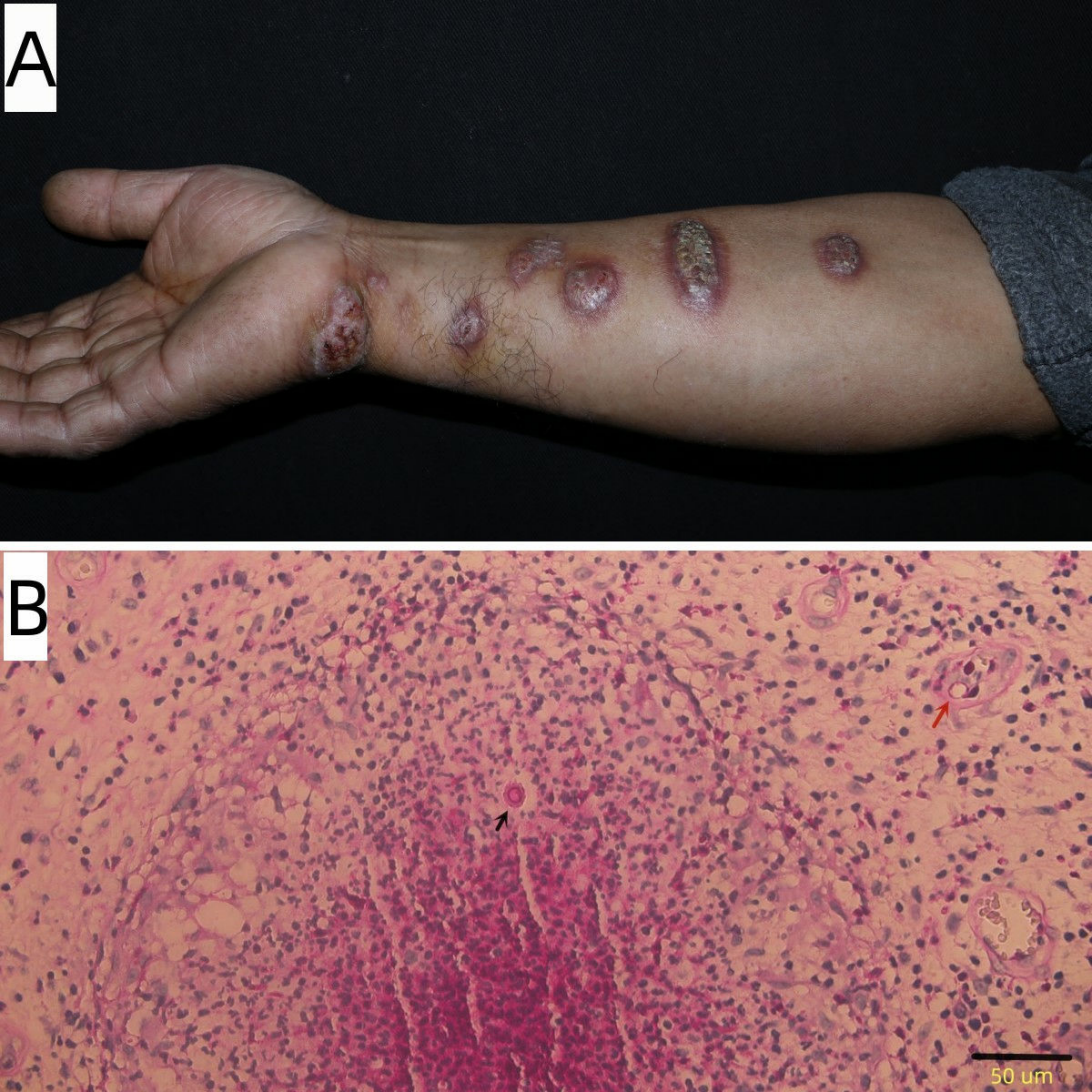
Linear distribution of nodules on the right upper extremity (**A**). Black arrow shows a round asteroid body, and red arrow shows a round yeast cell (B, PAS × 400).

A 56-year-old otherwise healthy male farmer presented to our dermatology clinic with a 3-month history of progressively increasing nodules on the right upper extremity. The patient indicated that nodular lesions developed approximately 2 weeks after a traumatic wrist injury during farming activities. Physical examination revealed several dull-red, crusted nodules distributed in a sporotrichoid and beaded pattern along the right upper extremity (Fig. 1). A biopsy of the lesions was performed for histopathological evaluation and fungal culture. Hematoxylin and eosin staining revealed diffuse inflammatory cell infiltration in the dermis, with multiple epithelioid cell granulomas containing central neutrophilic microabscesses. Periodic acid-Schiff (PAS) staining showed that yeast cells were present within the dermis; some yeast cells were encapsulated by a prominent layer of reddish material, with numerous neutrophils diffusely distributed in the surrounding area (Fig. 1B).

What is your diagnosis?

## ANSWER TO PHOTO QUIZ

The patient was diagnosed with lymphocutaneous sporotrichosis. Sporotrichosis is a subcutaneous mycosis caused by dimorphic fungi of the genus *Sporothrix* (order Ophiostomatales). Among the pathogenic species, *Sporothrix brasiliensis*, which is primarily transmitted through animal contact, exhibits high virulence and is predominantly endemic to South America, particularly Brazil. In contrast, *Sporothrix globosa* and *Sporothrix schenckii* follow classical saprophytic transmission pathways and demonstrate relatively lower virulence. *S. globosa* is primarily distributed across Asia, while *S. schenckii* has a global distribution (1). Definitive diagnosis of sporotrichosis requires culturing and subsequent characterization of *Sporothrix* species from patient-derived specimens. Fungal cultures were conducted in our mycology laboratory. Fungal cultures yielded colonies of *S. globosa*, which were confirmed by DNA sequencing (GenBank accession number PV203249). Based on these findings, a diagnosis of lymphocutaneous sporotrichosis was established. The lesions resolved completely following a 6-month course of systemic 10% potassium iodide (oral, 10 mL three times daily).

Histopathological examination of human samples may lack specificity and can only provide suggestive information for the diagnosis of sporotrichosis. Its histopathological features on skin biopsy are usually associated with granulomatous and pyogenic reactions and may be accompanied by epidermal hyperplasia (with or without ulceration), papillary spinous layer hypertrophy, hyperkeratosis, intraepidermal microabscesses, and the presence of fungal elements such as yeast cells and asteroid bodies (ABs) (2). ABs are histopathological structures characterized by a central eosinophilic core surrounded by radiating, homogeneous, refractile, eosinophilic, club-shaped projections. These structures represent a Splendore-Hoeppli reaction, which is a localized immunological host response to antigens from various infectious organisms, including fungi, bacteria, and parasites (3). In sporotrichosis, ABs are formed by the yeast cells and the surrounding antigen-antibody precipitation complex. According to the outline of the eosinophilic materials, the AB can be classified into five categories: stellate, club-shaped, ring-shaped, flower-shaped, and irregular (2).

AB is not a sporotrichosis pathognomonic structure, as it can occur in other infectious or granulomatous diseases (4), but in the case of central presence of yeast cells, combined with the clinical manifestations and medical history, it is a useful clue in the diagnosis of sporotrichosis (5). The sensitivity of histopathological tests for diagnosing cutaneous sporotrichosis remains limited due to the scarcity of fungal elements in the tissue samples (4). Fungal structures in tissues are observed in 18.00%–35.30% of cases, depending on the group of patients studied and the methodology employed (6). *Sporothrix* yeast cells exhibit a variety of morphologies, including round, oval, and cigar-shaped forms (7). The central spherical fungal structure within AB serves to decrease the volume-to-external surface ratio, thereby effectively minimizing the contact area (8). Itraconazole is widely regarded as the first-line treatment for sporotrichosis, owing to its well-documented efficacy, favorable safety profile, and convenient dosing regimen (9). Potassium iodide has been traditionally used in the treatment of sporotrichosis since the early 20th century. Nowadays, it is still used to treat cutaneous sporotrichosis due to its low cost (7).
